# A combination of extracellular matrix‐ and interferon‐associated signatures identifies high‐grade breast cancers with poor prognosis

**DOI:** 10.1002/1878-0261.12912

**Published:** 2021-02-19

**Authors:** Mara Lecchi, Paolo Verderio, Vera Cappelletti, Francesca De Santis, Biagio Paolini, Melissa Monica, Sabina Sangaletti, Serenella Maria Pupa, Marilena Valeria Iorio, Giulia Bianchi, Massimiliano Gennaro, Giovanni Fucà, Filippo De Braud, Elda Tagliabue, Massimo Di Nicola

**Affiliations:** ^1^ Bioinformatics and Biostatistics Unit Department of Applied Research and Technological Development Fondazione IRCCS Istituto Nazionale dei Tumori Milan Italy; ^2^ Biomarker Unit Department of Applied Research and Technological Development Fondazione IRCCS Istituto Nazionale dei Tumori Milan Italy; ^3^ Unit of Immunotherapy and Anticancer Innovative Therapeutics Department of Medical Oncology and Hematology Fondazione IRCCS Istituto Nazionale dei Tumori Milan Italy; ^4^ Pathology A Unit Department of Pathology Fondazione IRCCS Istituto Nazionale dei Tumori Milan Italy; ^5^ Molecular Immunology Unit Department of Research Fondazione IRCCS Istituto Nazionale dei Tumori Milan Italy; ^6^ Molecular Targeting Unit Department of Research Fondazione IRCCS Istituto Nazionale dei Tumori Milan Italy; ^7^ Medical Oncology Department Fondazione IRCCS Istituto Nazionale dei Tumori Milan Italy

**Keywords:** gene signature, high‐grade breast cancer, prognostic marker, tumor microenvironment

## Abstract

Breast cancer (BC) is a heterogeneous disease in which the tumor microenvironment (TME) seems to impact the clinical outcome. Here, we investigated whether a combination of gene expression signatures relating to both the structural and immune TME aspects can help predict prognosis in women with high‐grade BC (HGBC). Thus, we focused on a combined molecular biomarker variable that involved extracellular matrix (ECM)‐associated gene expression (ECM3 signature) and interferon (IFN)‐associated metagene (IFN metagene) expression. In 97 chemo‐naive HGBCs from the METABRIC dataset, the dichotomous ECM3/IFN (dECIF) variable identified a group of high‐risk patients (ECM3^+^/IFN^−^ vs other; hazard ratio = 3.2, 95% confidence interval: 1.5–6.7). Notably, ECM3^+^/IFN^−^ tumors showed low tumor‐infiltrating lymphocytes, high levels of CD33‐positive cells, absence of PD‐1 expression, or low expression of PD‐L1, as suggested by immune profiles and immune‐histochemical analysis on an independent cohort of 131 HGBCs. To make our results transferable to clinical use, we refined the dECIF biomarker using reduced ECM3 and IFN signatures; notably, the prognostic value of this reduced dECIF was comparable to that of the original dECIF. After validation in a new BC cohort, reduced dECIF was translated into a robust qPCR classifier for real‐world clinical use.

## Graphical abstract

This study investigates the combination of two relevant molecular signatures of the tumor microenvironment (TME) in identifying aggressive high‐grade breast cancers (HGBCs). The novel molecular biomarker described here highlighted patients with worst HGBC prognosis and a peculiar TME. Finally, to improve the potential of the identified biomarker in the clinic, we reduced the two original signatures and technically validated the biomarker's expression through a qPCR‐based assay.

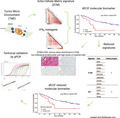

**Abbreviations**
BC: breast cancer
CI: confidence interval
dECIF: dichotomous ECM3/IFN
ECM: extracellular matrix
ER: estrogen receptor
FFPE: formalin‐fixed paraffin‐embedded
GEP: gene expression profile
H&E: hematoxylin and eosin
HGBC: high‐grade breast cancer
HR: hazard ratio
*k*: Cohen's kappa statistics
KW: Kruskal–Wallis
OS: overall survival
PgR: progesterone receptor
TIL: tumor‐infiltrating lymphocyte
TME: tumor microenvironment

## 1 Introduction

Breast cancer (BC) is the most common malignancy in women and a leading cause of cancer deaths worldwide [1]. Despite an overall improvement in BC clinical outcomes over the past 30 years, patients with high‐grade BC (HGBC) still experience a very low survival rate in the advanced setting, regardless of the molecular subtype [2]. Thus, the identification of patients with particularly poor prognosis in the early setting is mandatory to tailor a treatment‐intensification strategy, to minimize the chance of recurrence and eventually to ameliorate the clinical outcomes of HGBC patients. Most biomarkers routinely used for BC stratification are still ‘cancer‐cell oriented’, leaving out critical factors that contribute to the modulation of cancer progression, such as tumor microenvironment (TME) and antitumor immunity [3]. Focusing on the TME, we identified a specific extracellular matrix (ECM) gene expression pattern (ECM3) in ~ 30%–40% of BCs that classifies a biologically and clinically distinct group of tumors [4]. Genes identifying the ECM3 cluster encode for structural ECM proteins that are coordinately overexpressed by both stromal and BC cells. Notably, in patients with node‐negative, treatment‐naive early HGBC, we observed that the ECM3 gene expression pattern was associated with a higher relapse rate, independently of intrinsic molecular subtype [5]. Moreover, we have previously reported that, in treatment‐naive, node‐negative BC, a specific interferon (IFN) metagene (based on the expression levels of 21 genes) was associated with a distinct modulation of distant metastases risk according to the molecular subtype [5].

In this study, we combined the ECM3 signature and the IFN metagene in analysis of patients with early HGBC in order to investigate a novel molecular classifier with prognostic ability, which reflects the structural and immunological aspects of the TME. To optimize the clinical use of this novel prognostic tool, we additionally investigated the possibility of refining these signatures, with the goal of reducing their high dimensionality yet maintaining their good performance. Finally, to improve the chance of real‐world clinical use, we developed and technically validated a qPCR‐based assay that can easily identify early HGBC patients at high risk.

## 2 Materials and methods

### 2.1 Breast tumor case series

#### 2.1.1 METABRIC cohort

The METABRIC is a publicly accessible database containing more than 2000 gene expression profiles (GEPs) of primary breast tumors completed with clinico‐pathological and follow‐up data [6]. For this study, the subset of 97 chemo‐naive HGBC patient data were considered for prognostic evaluation based on ECM3 and IFN signatures.

#### 2.1.2 ECTO cohort

The ECTO case series is a subset of 131 HGBCs belonging to the TRANS‐ECTO1 cohort of 283 patients from the multicenter phase III study ECTO1 [7], for which GEP is available [8, 9]. Raw and processed data were uploaded to the Gene Expression Omnibus repository with ID GSE147472. The ECTO cohort gene expression data were used to reduce the ECM3 and IFN signatures, and residual RNA samples were used for a technical validation of the reduced signatures. Tissue sections from the 51‐patient ECTO subgroup recruited at Fondazione IRCCS Istituto Nazionale dei Tumori (INT) of Milan were used for a direct evaluation of the immune TME by IHC.

#### 2.1.3 INT cohort

The INT series includes 45 HGBCs obtained from women undergoing surgery at INT between 2008 and 2017. All procedures were performed in accordance with the Declaration of Helsinki. Samples remaining after diagnostic procedures were obtained from patients (with informed consent) and were used after approval by the institutional review board and a specific request to the Independent Ethical Committee of INT (INT 51/14). This independent cohort of patients was used for additional evaluation of the robustness of the reduced signatures.

The clinico‐pathological characteristics of patients belonging to each cohort are given in Table 1.

**Table 1:** Patient characteristics of each cohort. −, negative; +, positive.

Variable	METABRIC	ECTO	INT
	*N *= 97	*N *= 131	*N* = 45
	Median (IQR)	Median (IQR)	Median (IQR)
Age at diagnosis (yrs)	49 (62–70)	51 (43–58)	56 (48–71)
Tumor size (mm)	25 (18–32)	27.5 (21.5–31)^a^	22 (17–30)

	*N* (%)	*N* (%)	*N* (%)
ER status			
Negative	48 (49.5)	70 (53.4)	5 (11.1)
Positive	49 (50.5)	61 (46.6)	40 (88.9)
PgR status			
Negative	64 (66.0)	61 (46.6)	13 (28.9)
Positive	33 (34.0)	70 (53.4)	32 (71.1)
ECM3			
ECM3^−^	67 (69.1)	89 (67.9)	32 (71.1)
ECM3^+^	30 (30.9)	42 (32.1)	13 (28.9)
IFN			
IFN^−^	44 (45.4)	55 (42.0)	22 (48.9)
IFN^+^	53 (54.6)	76 (58.0)	23 (51.1)
ECM3/IFN joint			
ECM3^−^/IFN^+^	35 (36.1)	46 (35.1)	15 (33.3)
ECM3^−^/IFN^−^	32 (33.0)	43 (32.8)	17 (37.8)
ECM3^+^/IFN^+^	18 (18.6)	30 (22.9)	8 (17.8)
ECM3^+^/IFN^−^	12 (12.4)	12 (9.2)	5 (11.1)

^a^ Available on 104 pts.

### 2.2 RNA extraction and microarray hybridization

In the INT cohort, RNA was extracted using 4 × 10 µm sections from formalin‐fixed, paraffin‐embedded (FFPE) tissue blocks with the miRNeasy FFPE kit (Qiagen, Valencia, CA, USA) according to manufacturer's instructions. After quality control, RNA was hybridized to Affymetrix Clariom S chips, according to manufacturer's instructions, by the Integrative Biology Platform facility (Dipartimento di Ricerca applicata e sviluppo tecnologico [DRAST], Milan, Italy). Raw and processed data were uploaded to the Gene Expression Omnibus repository with ID 147471.

### 2.3 GEP‐based classification for ECM3, IFN, and PAM50

The ECM3 sample cluster was identified using the large average submatrix biclustering of 738 ECM genes [10]. The list of ECM genes was generated as previously described [4]. The expression of the IFN metagene was used to classify the patients into IFN^+^ and IFN^−^ groups using the 50th percentile of the IFN metagene distribution as cutoff [6]. The research‐based PAM50 subtype predictor was applied using the publicly available algorithm as described [11] and by performing median centering of the PAM50 genes.

### 2.4 Pathological assessment of tissue blocks and immunohistochemistry

Fifty‐one available tissue blocks from the ECTO were retrieved, and the hematoxylin and eosin (H&E)‐stained tissue sections of FFPE tumor specimens were reviewed by a certified pathologist to confirm tumor grade and to assess tumor‐infiltrating lymphocytes (TILs).

Grading was evaluated using the Nottingham grading system [12]. The average density of TILs in tumor areas was calculated semi‐quantitatively as the ratio of the area occupied by mononuclear cell infiltrates to the entire stromal area (% TIL = area occupied by mononuclear cells in tumor stromal/total stromal area) [13]. The pathologist was blinded to tumor molecular characteristics.

Immunohistochemical characterization of TILs and stromal TME was performed using PD‐L1, CD8 (C8/144B, monoclonal; Dako, Glosturp, Denmark) as surrogate marker for cytotoxic T lymphocytes, PD‐1 as surrogate marker for activated T lymphocytes (NAT105, monoclonal; Biocare Medical, Pacheco, CA, USA) and CD33 (PWS44, monoclonal; Leica Biosystem, Wetzlar, Germany) as surrogate marker for myeloid and histiocytic–monocyte cells.

Antigen retrieval was performed at high temperatures (96–98 °C) with different buffers (CD8: EDTA, 15 min; CD33: citrate, 15 min; PD‐1: citrate, 40 min; PD‐L1: citrate, 30 min), and antigen–antibody reaction was highlighted using a commercial detection kit (EnVsion Flex+; Dako). Expressions of CD8, CD33, PD‐1, and PD‐L1 were scored as percentage. PD‐L1 for tumor cells was scored as percentage value of positive tumor cells.

The percentage of TILs was dichotomized (low vs high) according to a threshold value of 10% [14]. The percentages of the other IHC markers were opportunely dichotomized according to the median value of the corresponding distribution.

Each marker was evaluated by two blinded pathologists (MM and BP), and any discrepancy was reviewed and discussed until 100% agreement was reached.

### 2.5 qPCR

Twelve genes included in the reduced ECM3 and IFN classifiers, and three housekeeping genes (*ACTB*, *RPLP1*, *GAPDH*) were quantified by qPCR using TaqMan assays that yielded short amplicons (< 84 bp) and with experimentally proven optimal performance on FFPE‐derived RNA (tested on five samples). All primers were obtained from Applied Biosystems (Foster City, CA, USA).

The complete list of TaqMan probes is reported in Fig. S1 (panel A).

qPCR assays were conducted in triplicate in a total volume of 15 µL, which included 7.5 µL of TaqMan Fast Universal PCR master mix (Applied Biosystems), 0.75 µL of TaqMan® Gene Expression Assay (Applied Biosystems), and 10 ng of template. The following cycling conditions were used: 20 s 95 °C, followed by 40 cycles of 1 s 95 °C and 20 s 60 °C, in a 7900HT Fast Real‐Time PCR System cycler (Applied Biosystems). Each plate contained 29 samples in triplicate along with blanks and a calibrator for controlling interplate variability. qPCR curves and *C*_q_ (quantification cycle) values were generated using the sds2.4 software (Thermo Fisher Scientific, Waltham, MA, USA). The experimental scheme is shown in Fig. S1B.

### 2.6 CIBERSORT analysis

CIBERSORT, a computational method for quantifying cell fraction from bulk GEPs that allows the composition of different immune cell types in a tissue sample to be estimated, was applied to ECTO dataset using the publicly available algorithm as described [15] (http://cibersort.stanford.edu/). Analyses were done with 100 permutations, enabling quantile normalization and default statistical parameters.

### 2.7 Statistical analysis

Different statistical approaches were applied to the available data from the three patient cohorts (e.g., METABRIC, ECTO, and INT) to cover the different aims of this work that range from the prognostic evaluation of the considered signatures, to the assessment of their association/correlation as well as concordance.

For the prognostic assessment of the considered signatures (METABRIC cohort), overall survival (OS) was defined as time from surgery to death from any cause. The pattern of OS was estimated using the Kaplan–Meier method, and the survival curves were compared using the log‐rank test. The roles of the considered variables in OS were assessed using Cox regression analysis by considering the putative better category as the reference one.

Analyses also considered the ‘ECM3/IFN joint’ variable obtained by combining the modalities of the two signatures involved in the following categories: (a) ECM3^+^/IFN^−^, (b) ECM3^+^/IFN^+^, (c) ECM3^−^/IFN^−^, and (d) ECM3^−^/IFN^+^, as well as in only two categories by considering the new variable dichotomous ECM3/IFN (dECIF variable, ECM3^+^/IFN^−^, and ‘other’, meaning not ECM3^+^/IFN^−^). The dECIF variable was also analyzed in terms of OS after adjustment for each of the clinical variables reported in Table 1, as well as for tumor intrinsic molecular subtypes assigned by applying the PAM50 subtype predictor, by using a bivariate Cox regression model.

In each cohort, the associations between dECIF and the considered clinical variables were assessed through Fisher's exact test or the nonparametric Kruskal–Wallis (KW) test for categorical or continuous variables, respectively.

Subsequently, the nonparametric KW test was used to analyze the association between dECIF and the expression of the *CD3*, *CD8,* or *CD33* gene (after normalizing to the expression of the *CD45* gene) in both the METABRIC and ECTO cohorts, as well as the quantification of cell fractions of interest from CIBERSORT data in the ECTO cohort. To this end, the expression levels of the genes encoding the CD3 complex components (i.e., CD3D, CD3E, CD3G, and CD247) were considered. Specifically, a principal component analysis approach was used to generate scores based on the expression of all of these genes, and the resulting first component was used as a surrogate of *CD3* expression level.

Fisher's exact test was used to evaluate the association between each IHC marker and dECIF in the subgroup of the 51 ECTO samples.

To reduce the original signatures of ECM3 and IFN, an *ad hoc* selection procedure was used based both on results from association and correlation analyses and previously acquired knowledge [4]. In particular, the first step involved evaluating the relationship between the signature status (i.e., negative or positive), and the expression level of each of the involved genes using the KW test by filtering according to the modulation pattern of genes in the original signatures. Subsequently, the pairwise correlation between significant genes included in each signature was investigated using Spearman's rank correlation coefficient (*r*_s_) and its 95% confidence interval (CI). Correlations showing that an *r*_s_ lower confidence limit of > 0.6 was defined as strong correlation [16]. Iteratively, genes that were mostly listed as strong correlations and that were deemed to be the most relevant (based on prior knowledge) were retained for the next step. Finally, a selection based on the Bonferroni correction was applied, to identify two reduced lists of significant and nonredundant genes. For each list, genes were opportunely combined in a logistic regression (multivariate) model (i.e., all subset analysis) [17] to obtain reduced signatures that resemble the original ones. Once dichotomized using the optimal cutoff (i.e., value maximizing the concordance metric), each reduced signature was compared with the original one. For this, the level of agreement was evaluated by estimating Cohen's kappa statistics (*k*) and its 95% CI. Each kappa statistic value was interpreted in a qualitative manner, adopting the Landis and Koch [18] classification criteria. The same approach was adopted for evaluating the patterns of concordance between the two involved assays (Affymetrix microarrays and qPCR) during the technical validation. The strength of association between the results obtained through the two different assays was also assessed using Spearman's correlation coefficient. All statistical analyses were carried out with sas (version 9.4; SAS Institute Inc., Cary, NC, USA) and r software (The R Foundation for Statistical Computing, c/o Institute for Statistical and Mathematics, Wien, Austria), by considering a significance level of alpha = 0.05.

## 3 Results

### 3.1 Analysis of the prognostic role of the ECM3 and IFN signatures in patients with HGBC

**Fig. 1:**
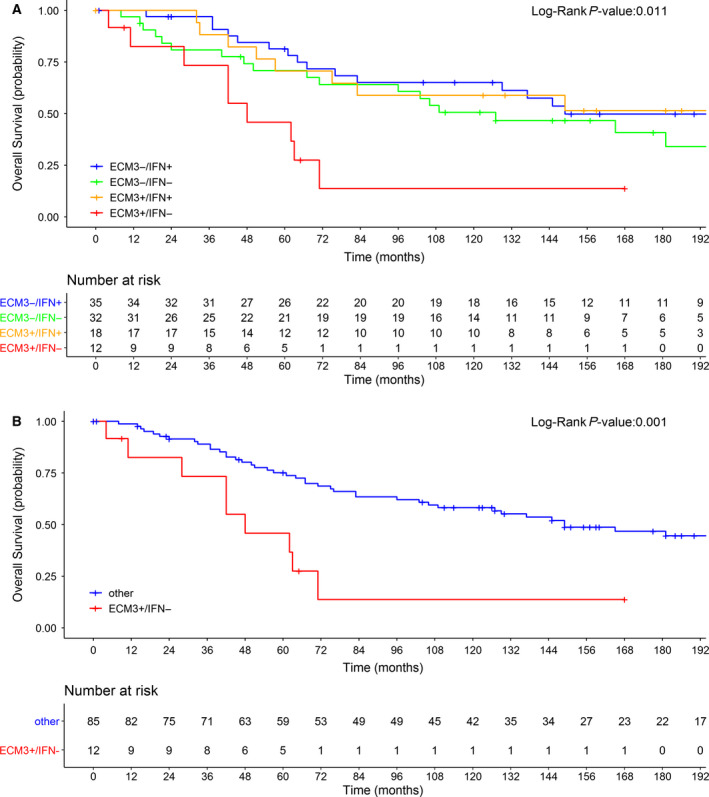
Sixteen‐year OS probability in untreated METABRIC patients based on ECM3/IFN and dECIF variables. (A) OS based on the joint ECM3/IFN variable categorized as ECM3^+^/IFN^−^, ECM3^+^/IFN^+^, ECM3^−^/IFN^−^, or ECM3^−^/IFN^+^. (B) OS according to the dECIF variable (ECM3^+^/IFN^−^ or ‘other’).

**Fig. 2:**
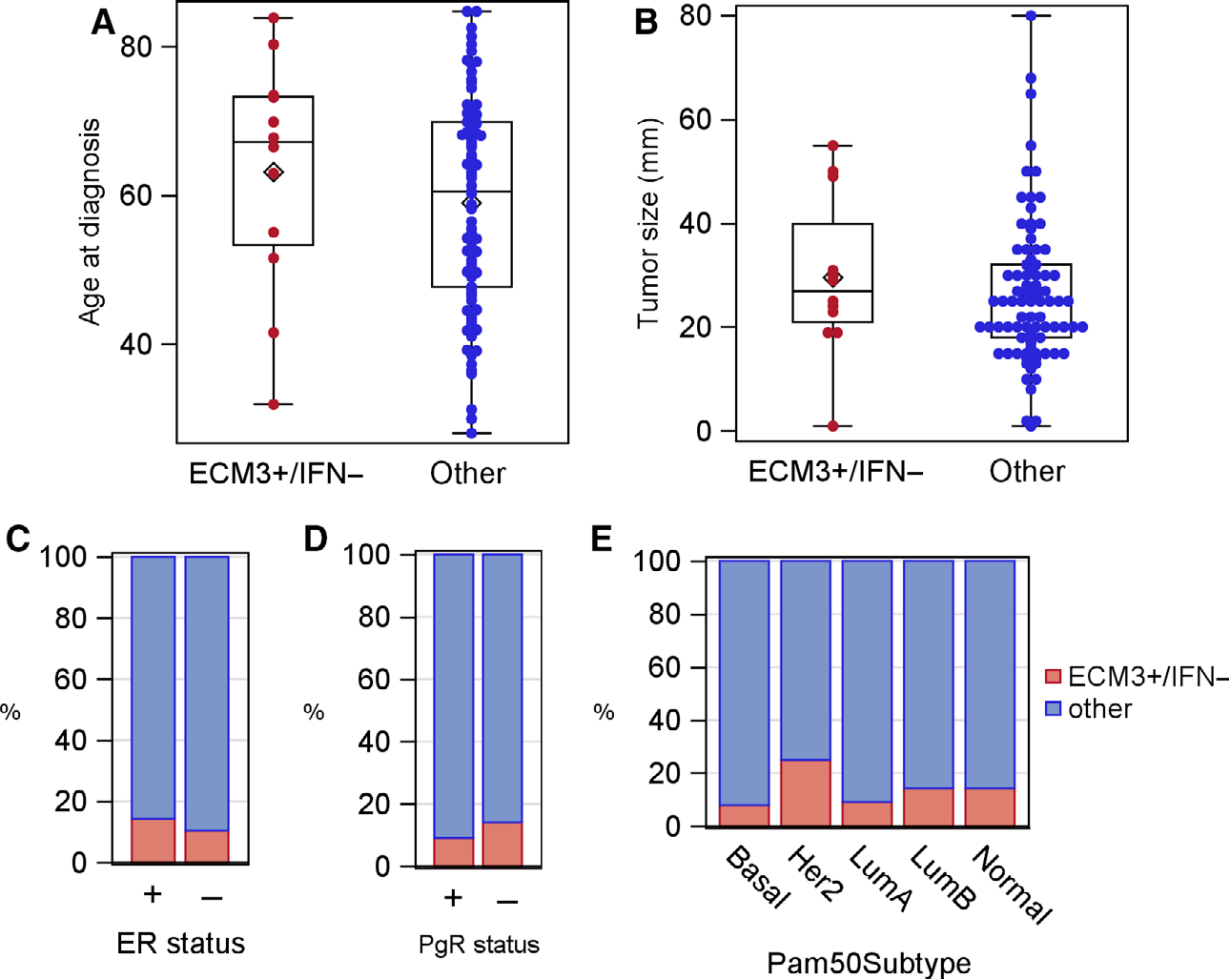
Distribution of the dECIF variable according to clinical variables and PAM50 in the METABRIC cohort. (A, B) Distributions of age at diagnosis (A) (KW test; *P* = 0.313) and tumor size (B) (KW test; *P* = 0.368) according to the dECIF variable. Each box indicates the 25th and 75th percentiles. The horizontal line inside the box indicates the median, and the whiskers indicate the extreme measured values. (C–E) The bar charts show the percentage of patients with BC classified as ECM3^+^/IFN^−^ or ‘other’, according to the ER status (C) (Fisher's exact test; *P* = 0.759, *n* = 49 positive, *n* = 48 negative), PgR status (D) (Fisher's exact test; *P* = 0.746, *n *= 33 positive, *n *= 64 negative), and PAM50 subtypes (E) (Fisher's exact test; *P* = 0.448, *n *= 38 basal, *n *= 16 Her2, *n *= 22 luminal A, *n *= 14 luminal B and *n *= 7 normal).

Using METABRIC database, we analyzed 97 chemo‐naive patients with HGBC to investigate the prognostic performance of the ECM3 and IFN signatures, either alone or jointly. At a median follow‐up time of 184 months [interquartile range (IQR) 130–228 months], the probability of OS for patients was 0.41 (95% CI: 0.30–0.52). Based on Cox regression analysis, ECM3, IFN, estrogen receptor (ER), progesterone receptor (PgR), and PAM‐50 predictors were not significantly associated with OS (Table 2). The only variables that retained statistical significance were age at diagnosis and tumor size. However, by considering the ECM3/IFN joint variable, we observed a statistically significant log‐rank test (*P* = 0.01; Fig. 1A). In particular, the ECM3^+^/IFN^−^ category identified BC patients with the worst prognosis with respect to other patients (Fig. 1A). We confirmed the result by classifying the patients into two categories (ECM3^+^/IFN^−^ vs other) of the dECIF variable, which gave an hazard ratio (HR) equal to 3.20 (95% CI: 1.52; 6.71; Fig. 1B), supporting the potentially clinical relevance of a ECM3/IFN joint variable in defining the most aggressive HGBCs. Notably, dECIF was not significantly associated with any other considered clinical variables (Fig. 2). We confirmed the prognostic role of the dECIF variable after adjustment for age at diagnosis (adjusted HR = 3.45; 95% CI: 1.61–7.39), tumor size (adjusted HR = 3.24; 95% CI: 1.52–6.87), ER status (adjusted HR = 3.19; 95% CI: 1.51–6.71), PgR status (adjusted HR = 3.40; 95% CI: 1.57–7.38), and PAM50 predictor (adjusted HR = 3.74; 95% CI: 1.71–8.15). These findings support the relevance of the TME characteristics, which are recapitulated by a ECM3/IFN joint variable, in identifying aggressive HGBCs.

**Table 2:** Variables considered in the univariate Cox analysis of OS. −, negative; +, positive.

Variable	HR	95% CI	*P*‐value
ECM3			
ECM3^+^	1.47	0.83; 2.61	0.191
ECM3^−^ ^a^	–		
IFN			
IFN^−^	1.59	0.94; 2.69	0.087
IFN^a^	–		
Age at diagnosis			
Continuous	1.03	1.00; 1.05	0.018
Tumor size			
Continuous	1.02	1.00; 1.04	0.013
ER status			
Negative	1.06	0.62; 1.82	0.827
Positive^a^	–		
PGR status			
Negative	1.00	0.57; 1.74	0.998
Positive^a^	–		
Pam50Subtype			
Basal	1.58	0.47; 5.34	0.464
Her2	2.62	0.73; 9.41	0.141
Luminal A	1.21	0.34; 4.26	0.768
Luminal B	2.46	0.67; 9.00	0.175
Normal^a^	–		
ECM3/IFN joint			
ECM3^+^/IFN^−^	3.62	1.58; 8.28	0.002
ECM3^−^/IFN^−^	1.31	0.69; 2.49	0.407
ECM3^+^/IFN^+^	1.10	0.49; 2.47	0.817
ECM3^−^/IFN^a^	–		
dECIF			
ECM3^+^/IFN^−^	3.19	1.52; 6.71	0.002
Other (not ECM3^+^/IFN^−^)^a^	–		

^a^ Reference category.

### 3.2 Characterization of the tumor microenvironment according to dECIF classification

**Fig. 3:**
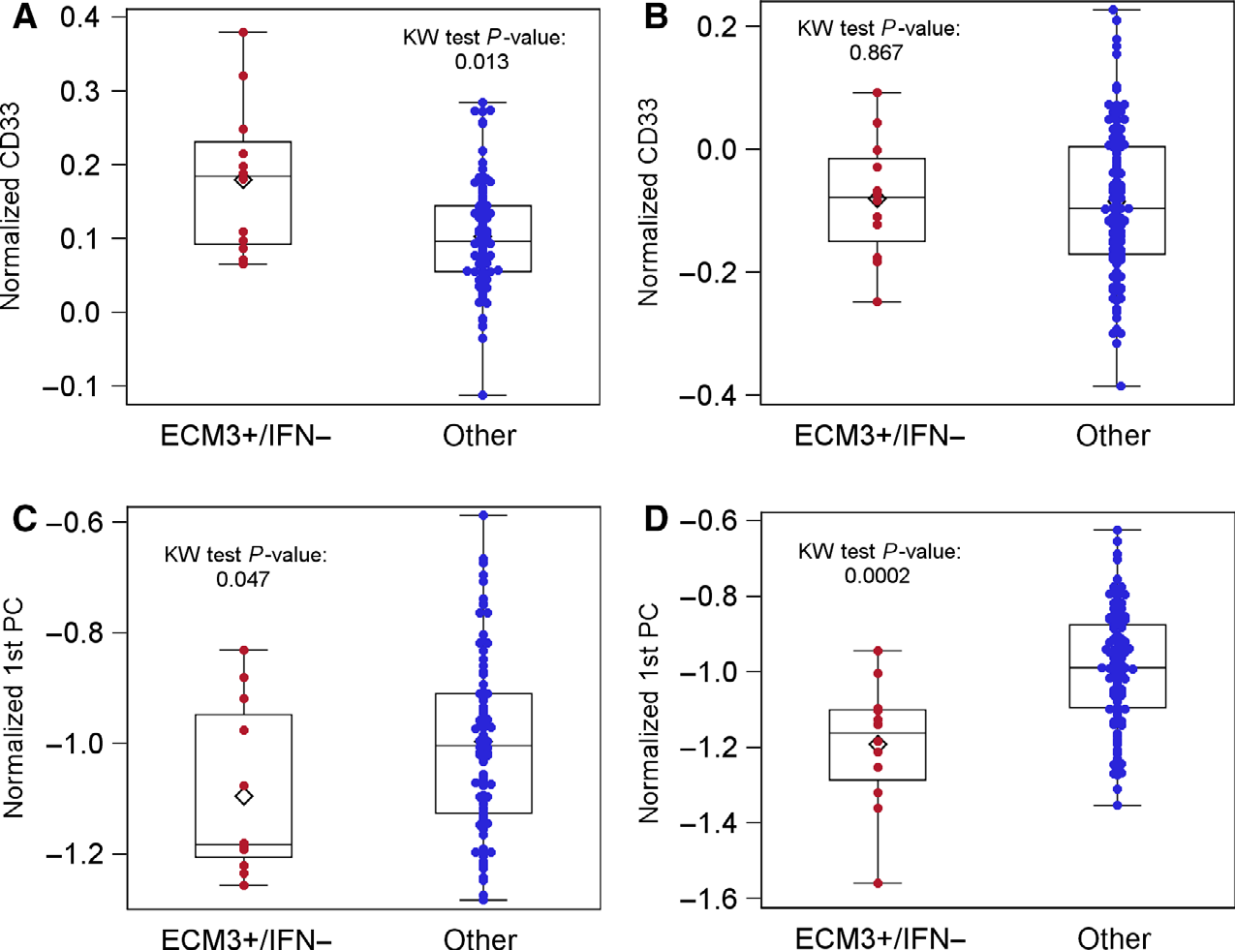
Association between normalized gene expression levels and the dECIF variable. (A, B) Distribution of gene expression levels of *CD33* according to the dECIF variable of the METABRIC (A) or ECTO cohorts (B). (C, D) Distributions of first principal component (1st PC) obtained from CD3‐subtype expression levels (CD3e, CD3g, CD3z, and CD247), according to dECIF of the METABRIC (C) or ECTO cohorts (D). Each box indicates the 25th and 75th percentiles. The horizontal line inside the box indicates the median, and the whiskers indicate the extreme measured values.

Based on the reported correlation between expression of the IFN metagene and T‐cell metagene [5], as well as the lack of enrichment for immune genes in the ECM3^+^ subgroup of BC patients [4], we investigated the immune TME by considering the ECM3 and IFN markers jointly. In the METABRIC cohort, we observed a statistically significant association between dECIF and *CD33* or *CD3* gene expression levels (Fig. 3). *CD33* was especially higher in the ECM3^+^/IFN^−^ samples as compared to the others (Fig. 3A), whereas the *CD3* level was lower (Fig. 3C). The same patterns of association were recorded in the ECTO cohort, although it was statistically significant only for *CD3* (Fig. 3B,D). No significant association was observed between *CD8* and dECIF in either the METABRIC cohort (KW test *P* = 0.974) or the ECTO cohort (KW test *P* = 0.667). We observed borderline associations between dECIF and Macrophages_M1 (KW test *P* = 0.054), B‐cell_memory (KW test *P* = 0.059), and T cells_CD8 (KW test *P* = 0.071) when analyzing the CIBERSORT data (Fig. S2).

**Fig. 4:**
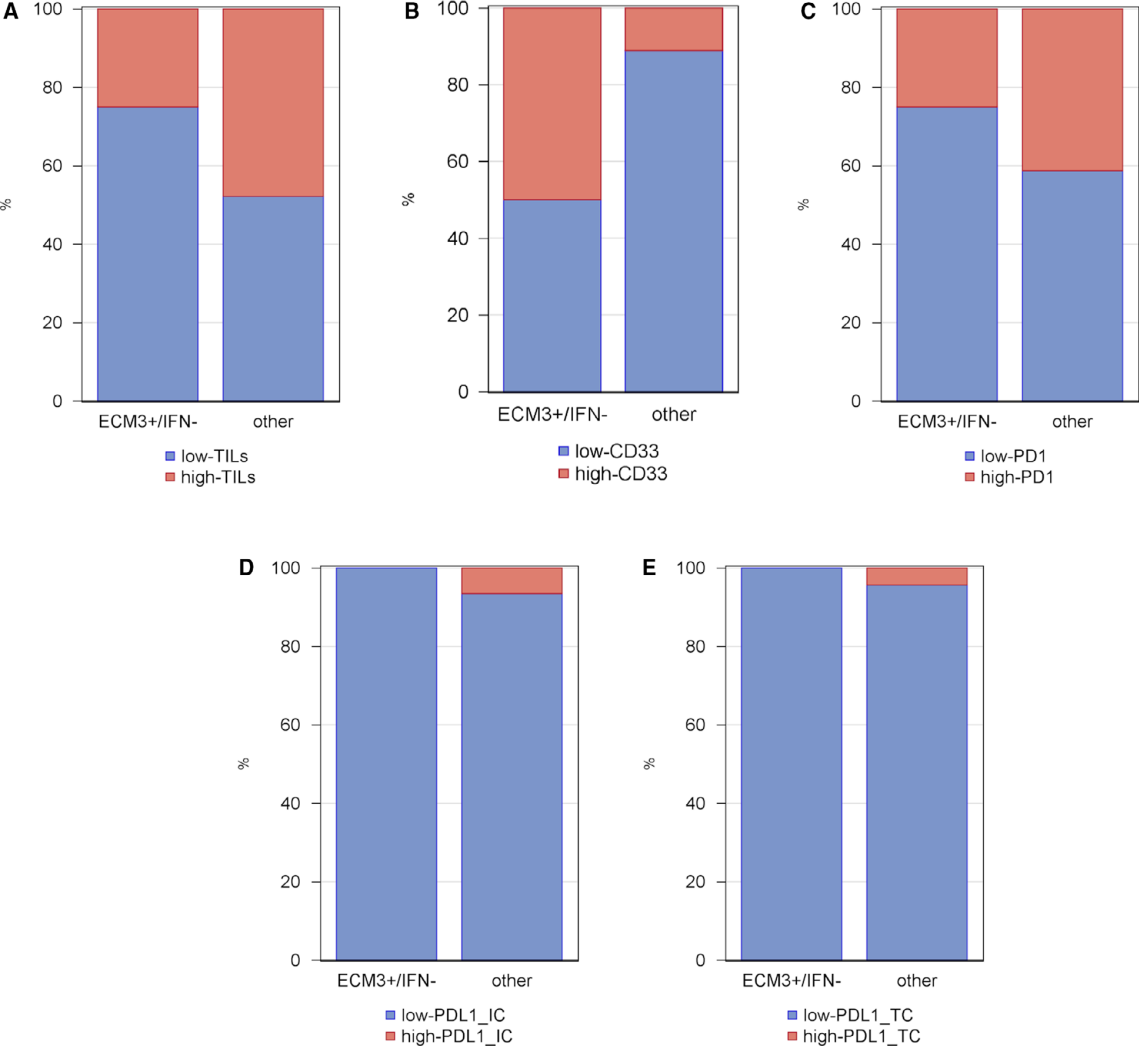
Percentage of patients with low or high IHC markers according to the dECIF variable Bar charts show the percentage of patients under (blue bar) or over (red bar) the threshold for each IHC marker ((A) TIL, (B) CD33, (C) PD1, (D) PDL1 on tumor infiltrating immune cells (IC) and (E) PDL1 on tumor cells (TC)), according to the dECIF variable. Thresholds of IHC markers correspond to the median of their distribution (threshold=0 for CD33, PDL1 IC, and PDL1 TC; threshold = 10 for TIL; threshold=20 for PD1).

We next aimed to verify the TME characteristics that emerged from analysis of the METABRIC and ECTO gene profiles. Given the lack of METABRIC tissue specimens, we examined 51 of the 131 ECTO available archival FFPE blocks for TILs on H&E stained sections and for immune markers by immunohistochemistry (IHC). Samples classified as ECM3^+^/IFN^−^ showed the highest percentage of cases in the lowest TIL infiltration category (Fig. 4A). Moreover, IHC analysis revealed a borderline significant difference in the proportion of myeloid CD33^+^ cells in the tumor subgroups (Fisher's exact test; *P* = 0.092; Fig. 4B), with the ECM3^+^/IFN^−^ subgroup comprising the lowest fraction of HGBCs classified in the CD33 low category, as well as the highest fraction of samples that did not express activation markers (such as PD‐1; Fig. 4C). PDL‐1 was not expressed (or almost not expressed) in any group in either tumor or immune cells (Fig. 4D,E). These findings strongly indicate that the ECM3^+^/IFN^−^ classification is useful for identifying a subgroup of HGBC cases that are characterized by a TME with a low number of nonactivated T cells as well as by myeloid cell infiltration.

### 3.3 Reduction of the ECM3 and IFN signatures

To promote a clinical application of the ECM3/IFN classification, we developed a more parsimonious model using ECTO molecular data, starting from the original high‐dimension ECM3 signature and IFN metagene, in order to obtain reduced signatures without a substantial loss of performance [22].

In a first step, 32 and 18 genes were significantly de‐regulated as in the original ECM3 or IFN signature, respectively (Fig. S3A,B). The iterative procedure started from the strong pairwise correlations (Fig. S4) and used the Bonferroni correction; it resulted in two short lists of genes (Table S1). Finally, we used multivariate analyses based on these selected genes to identify two signatures, of eight or four genes for ECM3 or IFN, respectively (Table 3). The patterns of agreement observed between the ECM3 and IFN status obtained through the original and the reduced signatures are reported in Table S2 for each of the three cohorts. According to the Landis and Koch criteria [18], both *k* statistic values obtained on the ECTO data were classified as almost perfect, with 0.83 (95% CI: 0.72; 0.93) for the ECM3 signature, and 0.94 (95% CI: 0.88; 1) for the IFN signature. To increase confidence in the robustness of our findings, we used an independent HGBC cohort (INT cohort) with comparable characteristics for validation of the results. The reduced signatures were used to analyze the status of patients originally classified as shown in Table 1. Importantly, the level of agreement between the ECM3 and IFN status obtained through the original and reduced signatures was confirmed, with *k*‐values of 0.90 (95% CI: 0.76; 1) for ECM3 and 1 (95% CI: 1; 1) for IFN (Table S2). Furthermore, an almost perfect agreement was observed even when the interchangeability was assessed on the METABRIC dataset, with *k*‐values of 0.98 (95% CI: 0.93; 1) for ECM3, and of 0.88 (95% CI: 0.78; 0.97) for IFN (Table S2).

**Table 3:** List of genes included in the ECM3 and IFN reduced signatures.

Signature	Gene	Number of genes
ECM3	BGN	8
	EFEMP2	
	ITGB5	
	NID2	
	PCOLCE	
	SERPINF1	
	SPARC	
	SPON1	
IFN	OAS1	4
	OAS3	
	MX1	
	IFI44L	

**Fig. 5:**
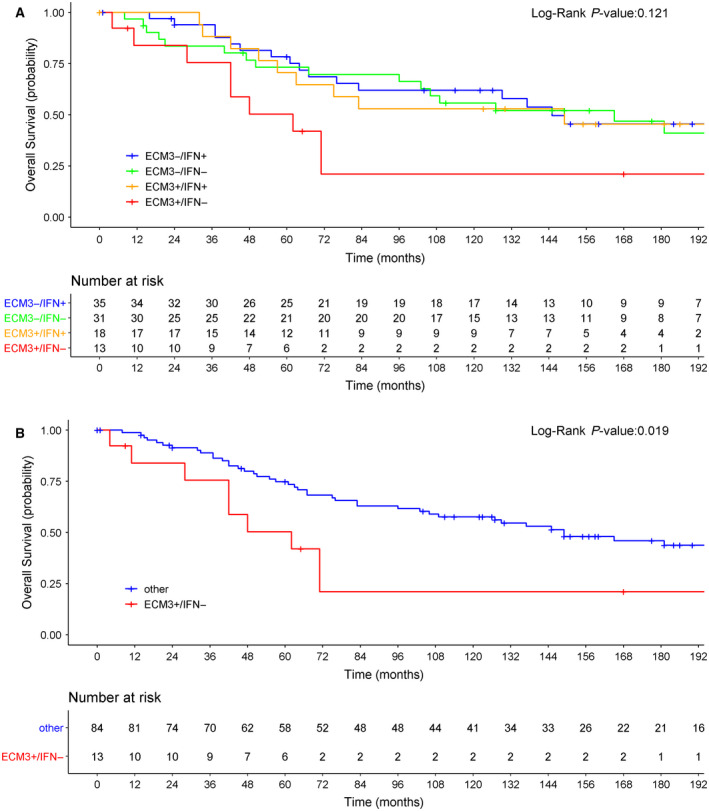
Sixteen‐year OS probability in untreated METABRIC patients using the ECM3/IFN joint and dECIF reduced variables. (A) OS according to the ECM3/IFN joint variable obtained from the reduced signatures and categorized as ECM3^−^/IFN^+^, ECM3^−^/IFN^−^, ECM3^+^/IFN^+^, or ECM3^+^/IFN^−^. (B) OS according to the two categories of dECIF variable obtained from the reduced signatures (ECM3^+^/IFN^−^ or ‘other’).

Taking into consideration the high levels of concordance observed for the METABRIC data, we performed the survival analysis starting from the ECM3/IFN status as defined through the reduced signature. A statistically significant HR equal to 2.60 (95% CI: 1.14; 5.89) was obtained from the ECM3/IFN variable for the specific contrast ECM3^+^/IFN^−^ vs ECM3^−^/IFN^+^ (Fig. 5). Moreover, the HR value observed for the dECIF variable (ECM^+^/IFN^−^ vs ‘other’) was statistically significant (HR: 2.32 95% CI: 1.12; 4.80; Table 4).

**Table 4:** Univariate Cox analysis results according to the ECM3/IFN status obtained from the reduced signatures. −, negative; +, positive.

Variable	*N*	%	HR	95% CI	*P*‐value
ECM3 reduced					
ECM3^+^	31	32.0	1.53	0.87; 2.69	0.145
ECM3^a^	66	68.0	–		
IFN reduced					
IFN^−^	44	45.4	1.33	0.78; 2.25	0.294
IFN^a^	53	54.6	–		
ECM3/IFN joint reduced					
ECM3^+^/IFN^−^	13	13.4	2.60	1.14; 5.89	0.023
ECM3^−^/IFN^−^	31	32.0	1.18	0.61; 2.26	0.626
ECM3^+^/IFN^+^	18	18.6	1.25	0.57; 2.75	0.573
ECM3^−^/IFN^a^	35	36.0	–		
dECIF reduced					
ECM3^+^/IFN^−^	13	13.4	2.32	1.12; 4.80	0.024
Other (not ECM3^+^/IFN^−^)^a^	84	86.6	–		

^a^ Reference category.

Because of the significant associations found in METABRIC cohort between dECIF and CD3 and between dECIF and CD33 (see Fig. 3A,C), the prognostic role of these genes was further investigated. By implementing a univariate Cox regression model, only CD3 (normalized levels × 10^2^) was found to be significantly associated with OS (HR: 0.97, 95% CI: 0.95; 0.99). Notably, the dECIF variable retained its significance even when adjusted for the CD3 levels (*dECIF* HR: 2.31, 95% CI: 1.10; 4.82, *CD3* HR: 0.97, 95% CI: 0.95; 0.99).

### 3.4 Toward a real‐world clinical use of the reduced ECM3 and IFN reduced signatures

To simplify its clinical application, we next developed a qPCR approach for attaining ECM3/IFN classification without the need of running GEPs. We used qPCR on 116 ECTO samples with good quality RNA to technically validate the ECM3 and IFN reduced signatures. For data normalization, we used the *RPLP1* gene expression level, which was found to be more stable than other housekeeping gene candidates, both alone and in combination with others. All genes analyzed with qPCR maintained the same modulation pattern (Figs S3 and S5). By evaluating the relationship between the gene expression from the qPCR assay and the original GEP, we obtained significant correlation coefficients ranging from 0.46 to 0.79. The regression line, together with the 95% CI for the ECM3 and INF classifiers [with *r*_s_ = 0.66 (*N* = 110) and *r*_s_ = 0.80 (*N* = 109), respectively], is shown in Fig. S6. Samples were tested for the ECM3/IFN status agreement based on the original and the qPCR signature by considering the dECIF variable (Table S3). A *k*‐value of 0.65 (95% CI: 0.43; 0.87) was obtained for the 109 samples, showing a substantial level of reproducibility.

## 4 Discussion

In this study, we provide evidence that a detailed investigation of the HGBC TME is crucial to identifying patients who will face rapid progression independently of intrinsic molecular subtype.

By taking into account the stroma complexity in terms of cellular and noncellular components, this study investigated the usefulness of combining two relevant molecular signatures, which reflect different aspects of the TME: the ECM composition, and infiltrating immune cells. By combining the analyses of both signatures in a subset of untreated HGBCs from METABRIC, we found that among patients already defined as high‐risk due to the pathologic grade, the ECM3^+^/IFN^−^ status was particularly associated with the worst prognosis in terms of OS. This underpins the clinical relevance of this new molecular marker in discriminating patients with the highest risk of disease progression after diagnosis, and who thus require an appropriate adjuvant treatment. dECIF is not associated with conventional clinical–pathological variables or to the PAM50 intrinsic molecular subtype in METABRIC (see Fig. 2) or in the other two analyzed cohorts (data not shown). In addition, the prognosis of ECM3^+^/IFN^−^ patients was poorer than that of patients classified in other subgroups, regardless of the standard clinical variables and PAM50. Of note, despite what is known in the overall BC population, the prognosis of HGBC patients does not seem to be influenced by the tumor intrinsic molecular characteristics. Hence, in these patients, the interplay between tumor cells and the TME emerges as the key factor for risk stratification and for optimizing treatment decisions.

The relevance of combining the ECM3 and IFN signatures for treatment selection is further supported by results obtained by investigating the immune TME according to the ECM3 and IFN markers jointly. Although ECM3 BCs express lower levels of immune genes than non‐ECM3 BCs [4], those classified as ECM3^+^/IFN^−^ could be considered peculiar ‘cold’ tumors. Indeed, our results from both immune gene expression and H&E/IHC investigations showed that ECM3^+^/IFN^−^ tumors were classified in the lowest nonactivated T‐cell category, corroborating the finding that ECM composition and organization are crucial for immune cell infiltration as well as for their function [19]. Recently, we demonstrated that in HGBCs, the ECM3 phenotype affects immune cell infiltration, thereby determining an immunosuppressive microenvironment in which myeloid cells contribute to promote tumor aggressiveness by inducing the epithelial‐to‐mesenchymal transition [20]. Accordingly, even though ECM3^+^/IFN^−^ HGBCs were the least immune‐infiltrated tumors, the relative percentage of myeloid CD33^+^ cells present in this subgroup was higher than in other subgroups.

Consistent with the distinctive immune TME in ECM3^+^/IFN‐ HGBCs, dECIF showed a significant association with worse OS even when CD3 expression level was considered. This result highlights the relevance of dECIF and supports the hypothesis that the evaluation of infiltrating lymphocytes is not sufficient for distinguishing the most aggressive tumors.

Based on these immunosuppressive functional and phenotypic features of TME, we expect that patients with ECM3^+^/IFN^−^ HGBCs might have a very low chance of responding to therapy with immune checkpoint inhibitors. Thus, in addition to being a prognostic marker, the differential expression of our new molecular signature could also represent a predictive biomarker of response to immunotherapy, allowing physicians to avoid the toxicity of ineffective treatments for ECM3^+^/IFN^−^ HGBC patients and to optimize the management of these expensive drugs. In this view, we focused on developing a simple assay based on a qPCR approach, which can be used with a good performance even on FFPE samples. In line with this, we refined the two molecular signatures to obtain a multigene predictor according to the principle of parsimony [21], which is essential not only for maintaining the robustness of the assay but also for making the result more directly transferable to the clinic. A qPCR‐based method set up to measure the ECM3 and IFN joint signatures could indeed provide an objective tool for identifying the most aggressive HGBCs, for which blockade of immune checkpoints is likely to be unsuccessful.

## 5 Conclusions

While the TME has previously been identified as a relevant marker for BC progression, its clinical usefulness has been limited by a lack of a separate evaluation of its single components, that is, ECM, resident immune, and stromal cells. We now show that, by combining two different stroma molecular signatures reflecting ECM and immune cells, a novel prognostic clinical applicable classifier could be generated. This marker is able to not only identify patients with HGBCs who have the worst prognosis but also to identify those requiring a more aggressive treatment as well as those who should be excluded from treatment with single‐agent PD‐1/PD‐L1 blockade due to the low expression of PD‐1/PD‐L1 and the high myeloid infiltration. Finally, the application of a qPCR‐based method to measure our new prognostic classifier makes this molecular tool particularly indicated for real‐world, clinical practice.

## Conflict of interest

The authors declare no conflict of interest.

### Peer Review

The peer review history for this article is available at https://publons.com/publon/10.1002/1878‐0261.12912.

## Author contributions

VC, PV, and ML conceived and performed experiments, analyzed data, interpreted results, and cowrote the manuscript; FDS interpreted results and cowrote the manuscript; BP and MM performed the pathological assessment of tissue blocks and immunohistochemistry; SS, SMP, MVI, and GF analyzed data and interpreted results; GB and MG provided surgical samples; FDB, ET, and MDN conceived experiments, analyzed data, interpreted the results, and cowrote the manuscript.

## Ethical approval

All procedures involving human samples were performed in accordance with the Declaration of Helsinki. Samples were obtained after patient’s informed consent and were used after approval by the institutional review board and a specific request to the Independent Ethical Committee of INT (INT51/14).

## Supporting information


**Fig. S1.** TaqMan assays used for qPCR gene expression measurements and experimental plan.
**Fig. S2.** Distribution of the considered CIBERSORT populations according to the dECIF variable.
**Fig. S3.** Univariate analysis of signatures genes for the ECTO cohort.
**Fig. S4.** Patterns of correlation between de‐regulated genes in ECM3 (panel A) and IFN (panel B) signatures.
**Fig. S5.** Univariate analysis of reduced signatures genes based on qPCR data.
**Fig. S6.** Correlation between Affymetrix and qPCR data.
**Fig.S7.** CD33^+^ mononuclear cells from the ECTO trial.
**Fig. S8.** CD33 ‐ mononuclear cells from the ECTO trial.
**Table S1.** Reduced lists of genes obtained from the selection procedure.
**Table S2.** Concordance for the ECTO, METABRIC, and INT cohorts.
**Table S3.** Concordance between ECM3 and IFN classifications in Affymetrix and qPCR assays for ECTO cohort.

## Data Availability

All data generated or analyzed during this study are included in this published article and its supplementary information files.
