# Supplementary material for: A combination of extracellular matrix‐ and interferon‐associated signatures identifies high‐grade breast cancers with poor prognosis
Source: Mol Oncol. 2021 Feb 19;15(5):1345–57. doi: 10.1002/1878-0261.12912 (PMC8096783; doi:10.1002/1878-0261.12912)
Supplement: Supplementary file 1 — Fig. S1. TaqMan assays used for qPCR gene expression measurements and experimental plan. Fig. S2. Distribution of the considered CIBERSORT populations according to the dECIF variable. Fig. S3. Univariate analysis of signatures genes for the ECTO cohort. Fig. S4. Patterns of correlation between de‐regulated genes in ECM3 (panel A) and IFN (panel B) signatures. Fig. S5. Univariate analysis of reduced signatures genes based on qPCR data. Fig. S6. Correlation between Affymetrix and qPCR data. Fig.S7. CD33+ mononuclear cells from the ECTO trial. Fig. S8. CD33 ‐ mononuclear cells from the ECTO trial. Table S1. Reduced lists of genes obtained from the selection procedure. Table S2. Concordance for the ECTO, METABRIC, and INT cohorts. Table S3. Concordance between ECM3 and IFN classifications in Affymetrix and qPCR assays for ECTO cohort. [file MOL2-15-1345-s001.docx]

**Supporting informations**

**Fig. S1. TaqMan assays used for qPCR gene expression measurements and experimental plan**

(A) List of genes included in the reduced signature and in TaqMan assays chosen for each gene. (B) Example of layout of a 96-well plate designed to detect a single gene in 29 samples. Each cell represents a technical triplicate, with the last three cells containing triplicates for blanks (e.g., no reverse transcriptase or water) and a triplicate from a calibrator (POOL) used to assess interplate variability.

**Fig. S2. Distribution of the considered CIBERSORT populations according to the dECIF variable**

Each box indicates the 25th and 75th percentiles. The horizontal line inside the box indicates the median, and whiskers indicate the extreme measured values.

**Fig. S3. Univariate analysis of signatures genes for the ECTO cohort**

(A,B) Distribution of expression levels of de-regulated genes of each original signature according to the ECM3 (A) or the IFN (B) status. Each box indicates the 25th and 75th percentiles. The horizontal line inside the box indicates the median, and whiskers indicate the extreme measured values. Red indicates positive status for ECM3 (A) or IFN (B), while blue indicates negative status for each.

**Fig. S4. Patterns of correlation between de-regulated genes in ECM3 and IFN signatures**

(A,B) Correlation patterns are shown for ECM3 (A) or IFN (B) signatures. Positive correlations are displayed in red and negative correlations, in blue. Color intensity and the size of the circle are proportional to the correlation coefficients. Of all the pairwise correlations, about 35% or 5% were classifiable as strong for ECM3 or IFN, respectively.

**Fig. S5. Univariate analysis of reduced signatures genes based on qPCR data**

(A,B) Distribution of qPCR expression levels of genes involved in each reduced signature according to ECM3 status (A) or IFN status (B). Each box indicates the 25th and 75th percentiles. Horizontal lines inside the boxes indicates the median, and whiskers indicate the extreme measured values. Red indicates distribution of genes according to positive (red) or negative (blue) status for 8 genes with ECM3 (A) or 4 genes with IFN (B).

**Fig. S6.** **Correlation between Affymetrix and qPCR data**

Scatter plots shows the correlation of the classifiers obtained through the Affymetrix assay (x-axis) and qPCR (y-axis). The regression line and the corresponding 95% CIs are also shown.

**Fig. S7. Representative immunohistochemistry staining of tumor samples classified as dECIF “ECM3+/IFN–” from patients enrolled in the ECTO trial.** Panel A: HE (20x). Panel B: rare CD8+ TILs (20x). Panel C: absence of PD1 + cells (20x). Panel D: presence of CD33 + mononuclear cells (20x).

**Fig. S8. Representative immunohistochemistry staining of tumor samples classified as dECIF “other” from patients enrolled in the ECTO trial.** Panel A: HE (10x). Panel B: abundant CD8+ TILs (10x). Panel C: presence of PD1 + cells (10x). Panel D: absence of CD33 + mononuclear cells (10x).

Figure S1

Figure S2


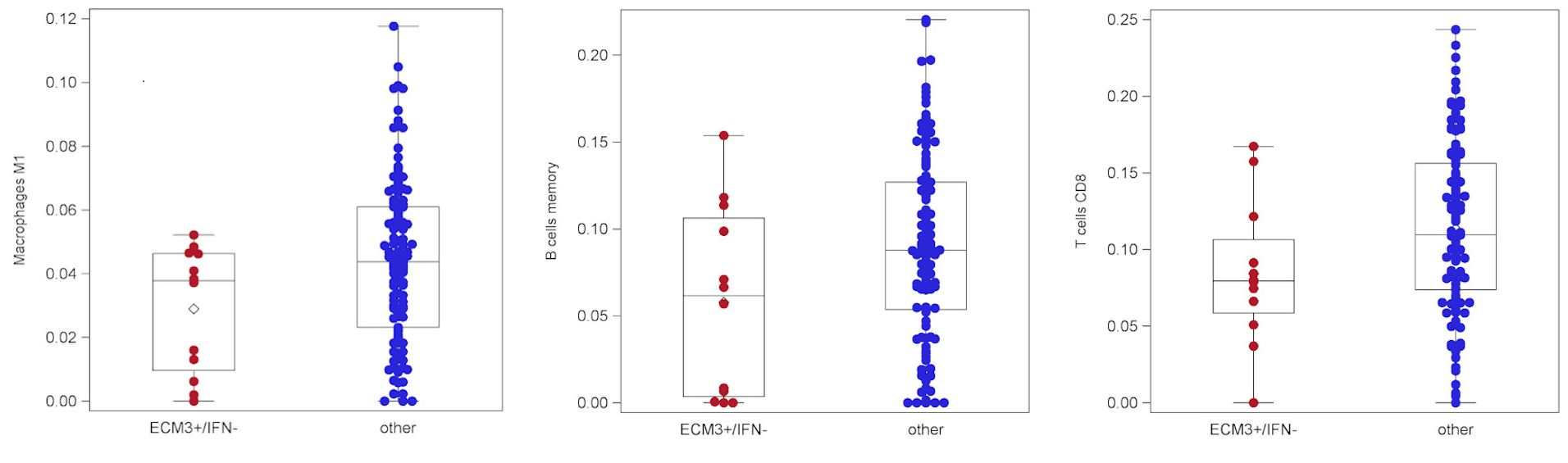


Figure S3


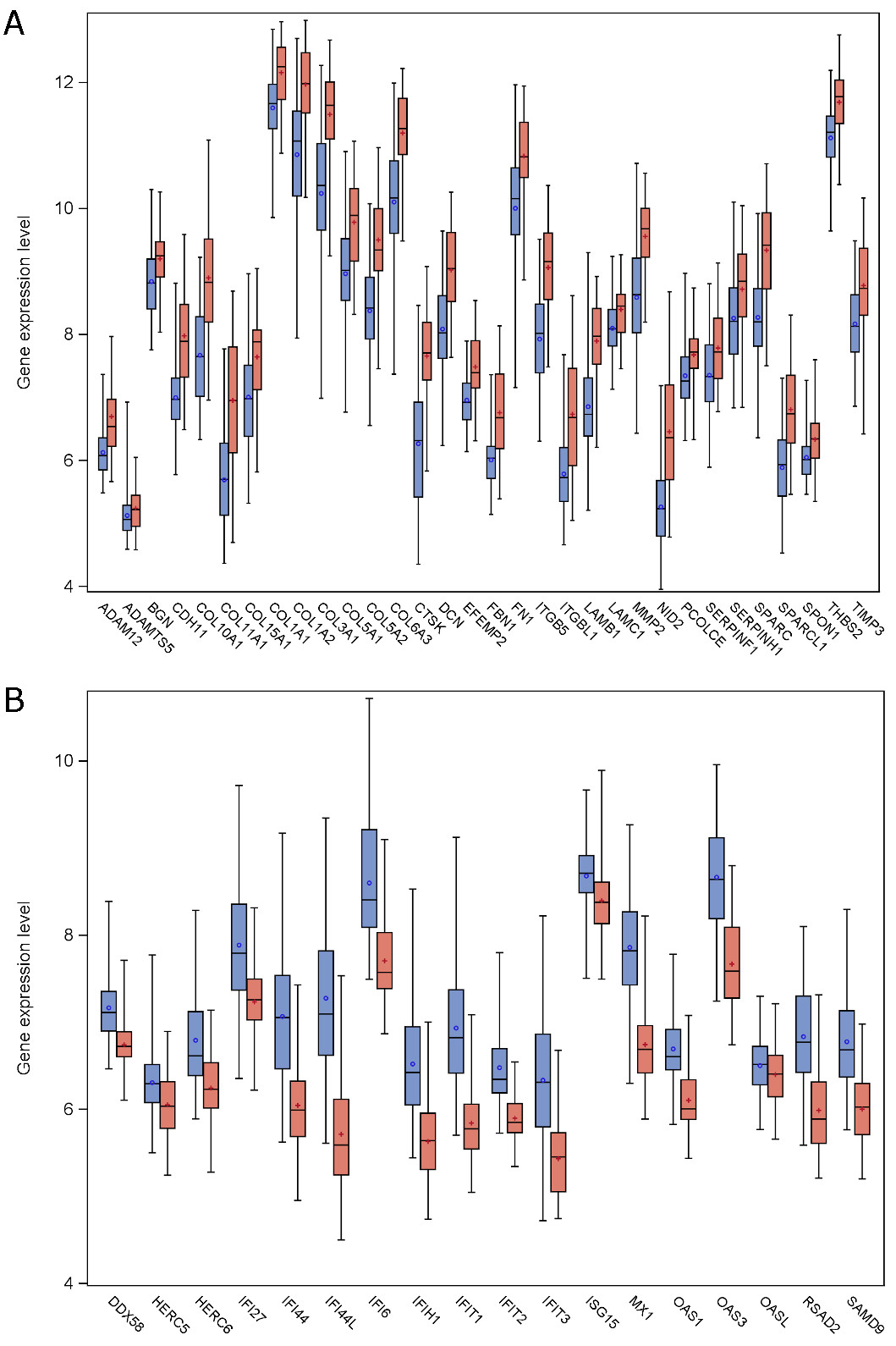


Figure S4


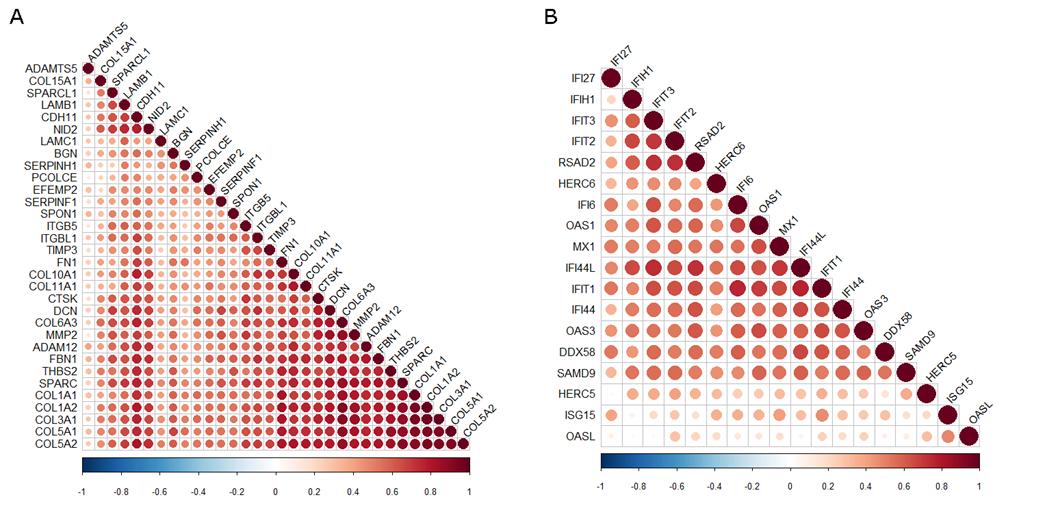


Figure S5


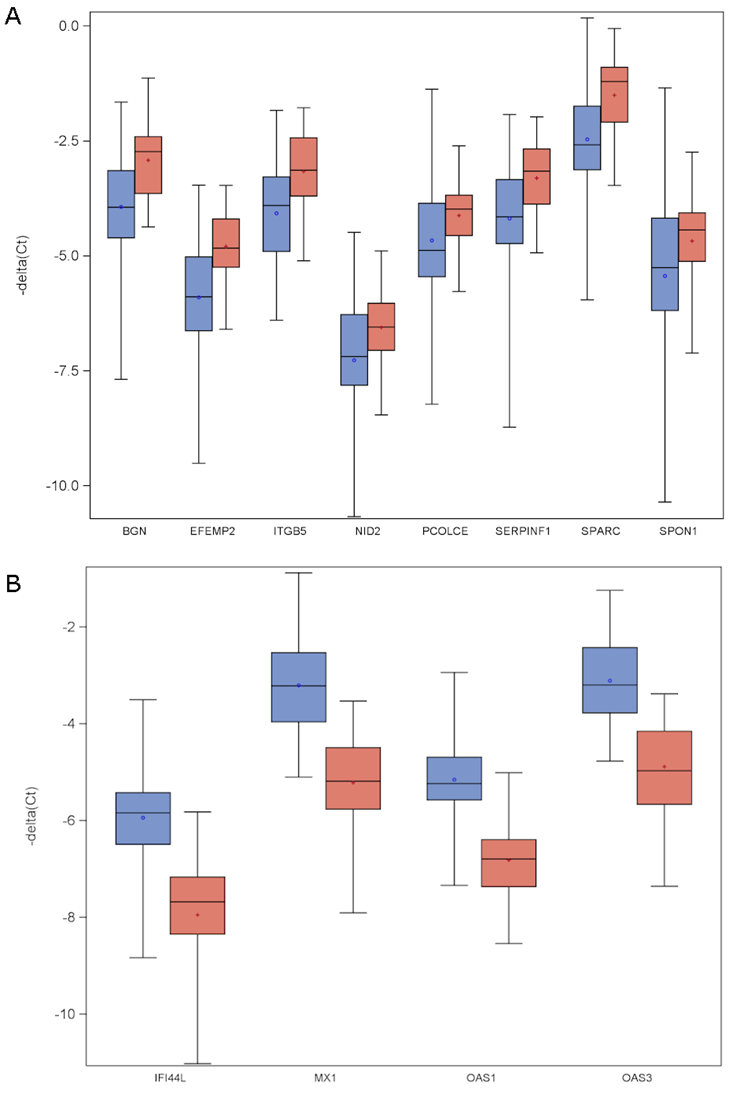


Figure S6


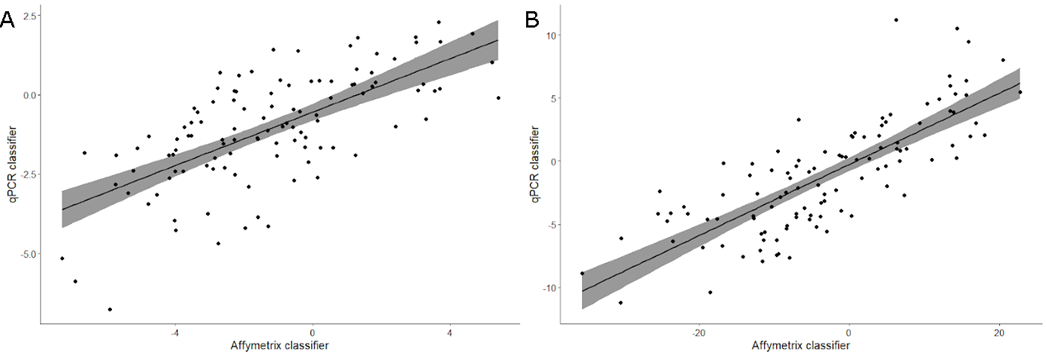

Figure S7

Figure S8

| Signature | Gene | Number of genes |
| --- | --- | --- |
| ECM3 | COL15A1 | 14 |
|  | COL1A2 |  |
|  | EFEMP2 |  |
|  | PCOLCE |  |
|  | SERPINF1 |  |
|  | SERPINH1 |  |
|  | TIMP3 |  |
|  | SPARCL1 |  |
|  | SPARC |  |
|  | BGN |  |
|  | NID2 |  |
|  | SPON1 |  |
|  | ITGBL1 |  |
|  | ITGB5 |  |
| IFN | DDX58 | 13 |
|  | HERC5 |  |
|  | HERC6 |  |
|  | IFI27 |  |
|  | IFI44 |  |
|  | IFI44L |  |
|  | IFI6 |  |
|  | IFIT2 |  |
|  | ISG15 |  |
|  | MX1 |  |
|  | OAS1 |  |
|  | OAS3 |  |
|  | SAMD9 |  |

**Table S1 Reduced lists of genes obtained from the selection procedure**

| **ECTO cohort** | | | |  | **METABRIC cohort** | | | |  | **INT cohort** | | | |
| --- | --- | --- | --- | --- | --- | --- | --- | --- | --- | --- | --- | --- | --- |
|  | ECM3 | | |  |  | ECM3 | | |  |  | ECM3 | | |
| ECM3 reduced | + | *–* | tot |  | ECM3 reduced | + | – | tot |  | ECM3 reduced | + | – | tot |
| + | 38 | 6 | 44 |  | + | 30 | 1 | 31 |  | + | 13 | 2 | 15 |
| - | 4 | 83 | 87 |  | - | 0 | 66 | 66 |  | - | 0 | 30 | 30 |
| tot | 42 | 89 | 131 |  | tot | 30 | 67 | 97 |  | tot | 13 | 32 | 45 |
|  |  |  |  |  |  |  |  |  |  |  |  |  |  |
|  | IFN | | |  |  | IFN | | |  |  | IFN | | |
| IFN reduced | + | – | tot |  | IFN reduced | + | – | tot |  | IFN reduced | + | – | tot |
| + | 73 | 1 | 74 |  | + | 50 | 3 | 53 |  | + | 23 | 0 | 23 |
| – | 3 | 54 | 57 |  | – | 3 | 41 | 44 |  | – | 0 | 22 | 22 |
| tot | 76 | 55 | 131 |  | tot | 53 | 44 | 97 |  | tot | 23 | 22 | 45 |
| –, negative; +, positive | | | |  | –, negative; +, positive | | | |  | –, negative; +, positive |  |  |  |

**Table S2 Concordance for the ECTO, METABRIC, and INT cohorts**

Concordance between ECM3 and IFN status obtained from the original signatures and the reduced signature, in the ECTO, METABRIC and INT cohorts.

|  | | |  | | | |
| --- | --- | --- | --- | --- | --- | --- |
|  | | | | *Affymetrix assay* | |  |
|  |  |  |  | ***dECIF*** | |  |
|  | | | | ***ECM3+/IFN–*** | ***Other*** | total |
| *qPCR assay* | ***dECIF*** | ***ECM3+/IFN–*** | | 9 | 6 | 15 |
|  |  | ***Other*** | | 2 | 92 | 94 |
|  |  | total | | 11 | 98 | 109 |

–, negative; +, positive; other, not ECM3+/IFN–

**Table S3 Concordance between ECM3 and IFN classifications in Affymetrix and qPCR assays for ECTO cohort**
